# Causal inference between pernicious anemia and cancers: a bidirectional two-sample mendelian randomization analysis

**DOI:** 10.1186/s12885-024-12354-y

**Published:** 2024-05-13

**Authors:** Bangwei Che, Shenglan Yuan, Hongyan Zhang, Jiancheng Zhai, Yang Zhang, Chuanchuan Wu, Kaifa Tang

**Affiliations:** 1 Department of Urology & Andrology, The First Affiliated of Guizhou University of Traditional Chinese Medicine, Guiyang, Guiyang 550001 China; 2 The First Clinical College, Guizhou University of Traditional Chinese Medicine, Guiyang, 550001 China; 3 Physical examination center, The First Affiliated of Guizhou University of Traditional Chinese Medicine, Guiyang, 550001 China

**Keywords:** Pernicious Anemia, Vitamin B12 deficiency, Pan cancer, Mendelian randomization, Cancer genetics

## Abstract

**Background:**

Observational study investigated the association between pernicious anemia (PA) and cancers. However, with the exception of gastric cancer, the results are mostly contradictory. The purpose of this study was to investigate the potential causal relationship between PA and cancers through bidirectional two-sample Mendelian randomized (MR) analysis.

**Methods:**

The European sample FinnGen project provided the genetic summary data for PA and 20 site-specific cancers. This bidirectional two-sample MR design mainly used the inverse variance weighting (IVW) method to evaluate the causal relationship between PA and cancer risk. Benjamini-Hochberg correction was performed to reduce the bias caused by multiple tests.

**Results:**

Our study shows that there was a causal relationship between PA and gastric cancer, prostate cancer, testicular cancer and malignant melanoma of skin, and there was a reverse causal relationship between prostate cancer or gastric cancer and PA (*P* < 0.05). After Benjamini-Hochberg correction test, there was still a causal correlation between PA and gastric or prostate cancer (*P’* < 0.05), while there was only an implied causal association between PA and testicular cancer and malignant melanoma of skin (*P*’> 0.05). There was still a reverse causal relationship between gastric cancer and PA (*P*‘< 0.05), while prostate cancer shows an implied reverse causal relationship(*P*’> 0.05). In addition, MR-Egger and MR-PRESSO tests showed no significant horizontal pleiotropy.

**Conclusions:**

PA may be genetically associated with testicular cancer, prostate cancer, gastric cancer, and malignant melanoma of skin.

**Supplementary Information:**

The online version contains supplementary material available at 10.1186/s12885-024-12354-y.

## Introduction

Pernicious anemia (PA) is an autoimmune disease, mainly due to autoantibodies targeting and destroying gastric parietal cells, resulting in vitamin B12 deficiency caused by a lack of internal factors [1]. This condition is often mistaken for simple vitamin B12 deficiency, but it specifically refers to the vitamin B12 deficiency caused by gastric atrophy and/or the lack of intrinsic factor [2]. PA is the end stage of autoimmune gastritis, a disease characterized by immune-mediated damage to gastric parietal cells, accompanied by gastric body atrophy and the absence of intrinsic factor.

A critical component of PA’s pathophysiology involves the destruction of the oxyntic mucosa, resulting in the subsequent emergence of hypo- and achlorhydria, as well as elevated circulating gastrin levels. Hypergastrinemia, a defining characteristic of PA, plays a significant role in the development of gastric neuroendocrine tumors (NETs) [3]. Gastrin, a peptide hormone, stimulates the growth and function of gastric cells, particularly endocrine cells. In the setting of PA, persistent hypergastrinemia can promote the proliferation of gastrointestinal chromaffin cells, precursors to NETs. Type I gastric NETs, also known as gastrinomas, are a subtype strongly linked to hypergastrinemia. These tumors are typically numerous, small, and located in the gastric mucosa or submucosa. They are thought to arise from the hyperplasia of enterochromaffin-like cells in response to chronically elevated gastrin levels. Consequently, individuals with PA, who often exhibit persistently elevated gastrin levels, are at an increased risk of developing type I gastric NETs [4]. The clinical management of PA patients should therefore include regular surveillance for the early detection of gastric NETs. This longstanding understanding has also contributed to the belief that patients with PA face a higher risk of gastric cancer [5, 6].

According to genetic epidemiological research, the genome of an individual plays a major role in determining their susceptibility to autoimmune diseases [7]. Therefore, it is generally believed that autoimmune diseases may lead to the development of cancer [8, 9]. In addition to gastric cancer, observational studies have shown inconsistent or missing evidence for the association between PA and other cancers [10, 11]. On the one hand, this may be due to the fact that PA is usually asymptomatic, which leads to an underestimation of the true prevalence of PA diagnosis and complications [12]. On the other hand, observational studies could be skewed by reverse causality and confounding variables [13]. This implies that in order to systematically evaluate the relationship between PA and cancer risk, genetic causal association analysis is required.

The Mendelian randomization (MR) study uses single nucleoside polymers (SNPs) as instrumental variables (IVs) to study the association between disease / exposure factors and disease, which can effectively solve the confounding and reverse causality of traditional observational studies, so it is regarded as a complementary strategy for randomized controlled trials [14]. Thus, in order to ascertain the causal relationship between PA and 20 site-specific cancers, this study employed a bidirectional two-way MR design.

## Methods

### Study design

**Fig. 1:**
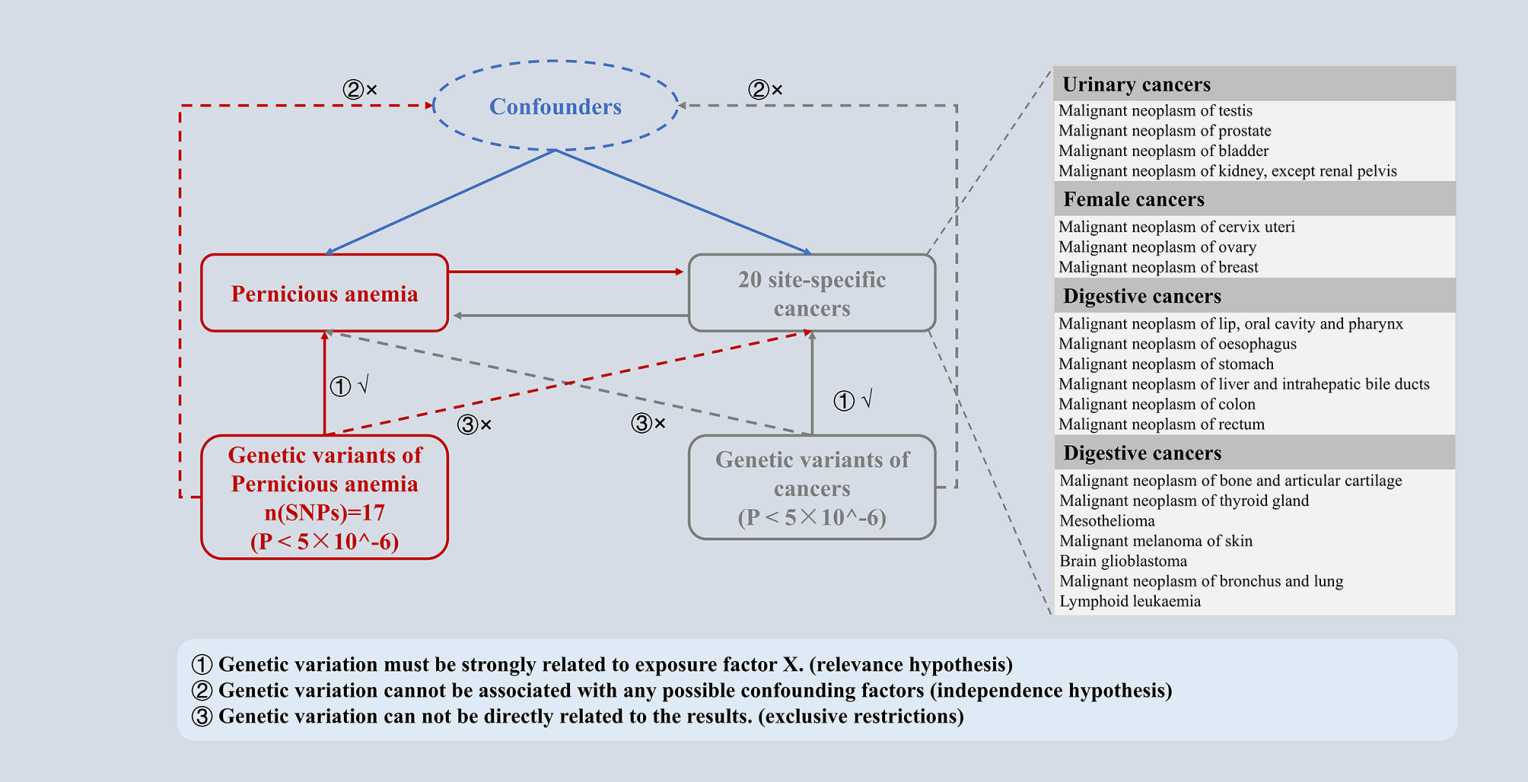
Overview of the study design in this bidirectional MR study

PA and cancer were assessed as causally related using a bidirectional two-sample MR analysis. Three fundamental presumptions served as the foundation for the MR study: (I) Relevance hypothesis, genetic IVs must be strongly related to PA (cancers). (II) Independence hypothesis, the selected IVs cannot be associated with confounding factors. (III) Exclusive hypothesis, IVs can only affect cancer (PA) risk through PA (cancer). In this study, the direction of the causal relationship between PA and cancer was further determined by a two-way MR design, and the potential reverse causal relationship was determined (Fig. 1).

### Exposure / outcome data source

PA and cancer data, including publicly summarized statistics of 21 genome-wide association studies (GWAS), were used in this study from the European sample FinnGen Project R5 (https://www.finngen.fi/en) (Supplementary Table 1). In order to minimize population stratification bias, the exposure and outcome cohorts were restricted to participants of European descent, and the cancer GWAS data excluded cases of other cancers.

This study does not require institutional review committee ethical approval because all FinnGen Project studies have been approved by the local institutional review committee and ethics committee.

### Instrumental variable selection and statistical analysis

For univariate two-sample MR analyses, the reference/alternative alleles were first examined for coordination between exposure and outcome. SNPs were limited by linkage disequilibrium (LD, r2 equilibrium = 0.001, and window size = 1000 kb). SNPs associated with PA (cancer) that have suggestive genome-wide significance (*P* < 5 × 10^-6) were employed as IVs for the disease because PA SNPs found by GWASs rarely reach the level of genome-wide significance (*P* < 5 × 10^-8). IVs associated with confounding factors of outcome were removed by examining the secondary phenotype of each SNP on the PhenoScanner. For the statistically significant IVs, F statistics are further calculated to verify whether they were strong tools [15, 16].

The Inverse variance weighted (IVW) were the main MR methods in this study. Benjamini-Hochberg correction was performed to reduce the bias caused by multiple tests. When IVW *P* < 0.05, but Benjamini-Hochberg corrected *P* > 0.05, the correlation was considered suggestive. On the contrary, if the Benjamini-Hochberg corrected *P* < 0.05, it was considered significant. MRlap was used to detect potential sample overlap and its impact on result bias [17]. MR-PRESSO was used to detect the existence of horizontal pleiotropy and to eliminate abnormal SNP (outliers) and estimate the corrected results (Supplementary Table 3). The MR-Egger intercept test was used to investigate the presence of directional pleiotropy in the effect estimates (*Pmr* < 0.05 indicating its existence), and the MR-Egger adjusted effect estimates, which accounts for average directional pleiotropy, were also reported. Cochran’s Q was used to test heterogeneity (pval < 0.05 indicating heterogeneity). In addition, leave-one-out was used to determine whether the estimated value is driven by a single SNP. Through a search on the PhenoScanner platform to determine the pleiotropy between PA-related SNPs and cancer. As PA and cancer phenotypes were binary, the effect estimates (log odds ratio) were converted by multiplying 0.693 (ln2), which emphasizes causal correlation and weakens causal effect size. The results could be explained by the effect of doubling the risk of PA on the risk of cancer. In the opposite direction, it showed the effect of doubling the risk of cancer on the risk of PA [18, 19].

All data processing, MR analysis and mapping are carried out in R software (version 4.3.1). The main software packages used include “TwoSampleMR”, “MRlap”, “dplyr”, “tidyverse”, “grid”, “forestploter”, “cmplot” and “MRPRESSO”. *P* < 0.05 was considered statistically significant.

## Results

### Selection of SNPs: PA IVs

We finally identified 17 independent SNPs significantly associated with PA as tool variables. Each SNP’s F statistical value was more than 10, which rules out any possibility of weak instrument bias (Supplementary Table 2). Based on the PhenoScanner search results, except for rs28407950 (cervical cancer) and rs17656368 (skin cancer), none of the other 15 IVs has 20 cancer-related SNPs. Subsequently, after removing the SNPs with horizontal pleiotropy, the final IVs were obtained.

The Leave-one method did not detect high-impact SNPs in any of the tests (Supplementary Figs. 1, 4, 7, and 10). MRlap method showed that the results of this study were not affected by sample overlap. There was no difference between MR-Egger intercept and zero, indicating that there was no directional pleiotropy. The heterogeneity between IVs was not detected by the Cochran Q test in this study (*P* > 0.05).

### Causal effects of PA on urinary cancers

**Fig. 2:**
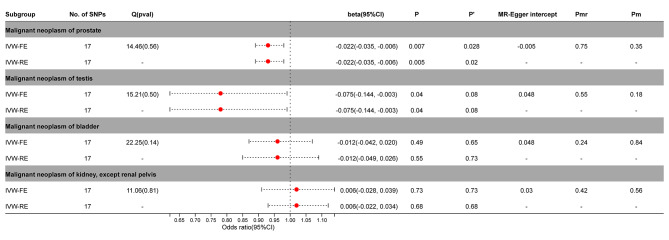
Association between genetic susceptibility to pernicious anemia and urinary cancers. IVW-FE; Inverse Variance Weighted Fixed Effects estimation method; IVW-RE; Inverse Variance Weighted Random Effects estimation method; beta, the average change (when exposed to the variable) in the outcome per 2-fold increase; SNP, single nucleotide polymorphism; 95%CI, 95% Confidence Interval; P, The IVW estimated P-value; P’, The IVW estimated P-value adjusted by the Benjamin-Hochberg method; Q(pval), Cochran Heterogeneity Test P-value; Pmr, MR–Egger P-value; Pm, MRlap P-value; -, Not applicable

In the MR analysis of PA and urinary tumors, the fixed effect / random effect IVW (IVW-FE / IVW-RE) model showed PA and testicular cancer (IVW-FE, beta = -0.075; 95%CI = -0.144, -0.003; *P* = 0.04; IVW-RE, beta = -0.075; 95%CI = -0.144, -0.003; *P* = 0.04) or prostate cancer (IVW-FE, beta = -0.022; 95% CI = -0.035, -0.006; *P* = 0.007; IVW-RE, beta = -0.022, 95%CI = -0.035, -0.006; *P* = 0.005) was a significant causal relationship between risks. The results of Benjamini-Hochberg correction test showed that there was still a significant causal relationship between prostate cancer and PA (*P’* <0.05). And the causal relationship between PA and testicular cancer was suggestive only (*P’* >0.05). No significant causal relationship was observed between PA and bladder cancer or renal cell carcinoma (*P* > 0.05) (Fig. 2, Supplementary Figs. 2–3).

### Causal effects of PA on female cancers

**Fig. 3:**
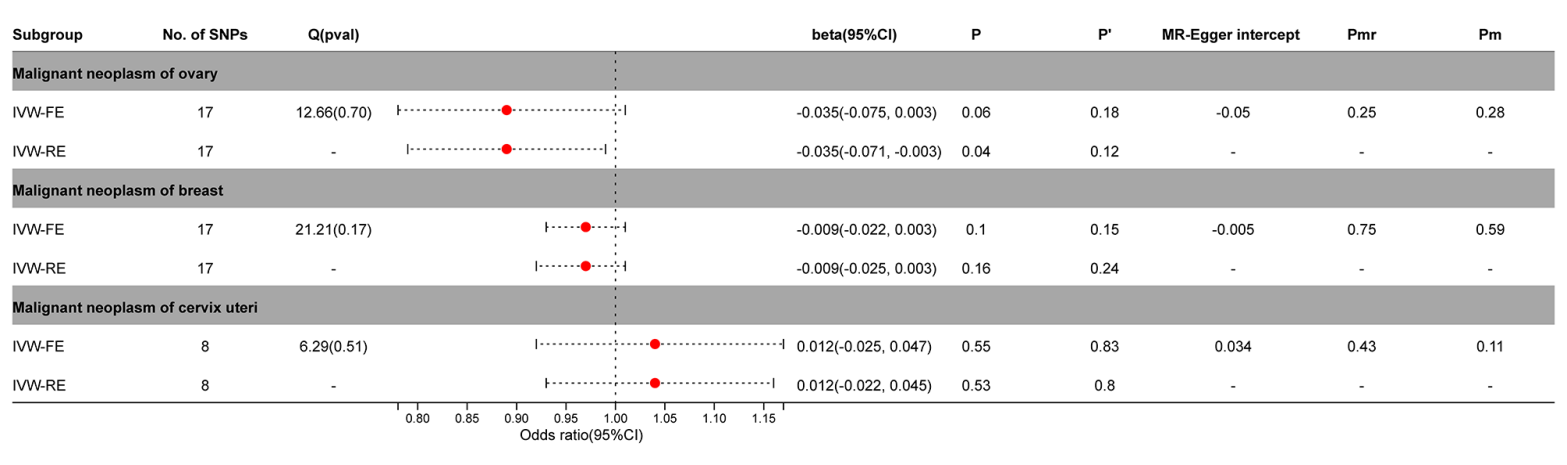
Association between genetic susceptibility to pernicious anemia and female cancers. IVW-FE; Inverse Variance Weighted Fixed Effects estimation method; IVW-RE; Inverse Variance Weighted Random Effects estimation method; beta, the average change (when exposed to the variable) in the outcome per 2-fold increase; SNP, single nucleotide polymorphism; 95%CI, 95% Confidence Interval; P, The IVW estimated P-value; P’, The IVW estimated P-value adjusted by the Benjamin-Hochberg method; Q(pval), Cochran Heterogeneity Test P-value; Pmr, MR–Egger P-value; Pm, MRlap P-value; -, Not applicable

After adjustment by MR-PRESSO, SNPs with horizontal pleiotropy in cervical cancer (rs9270535, rs140650994, rs79132259, rs75973258, rs7310615, rs151234, rs35056955, and rs73597298) were removed. Breast cancer or cervical cancer did not significantly correlate with PA (*P* > 0.05). In ovarian cancer, the IVW-FE model (beta =-0.035; 95%CI = -0.075, 0.003, *P* = 0.06) showed no significant causal relationship, while the IVW-RE model (beta =-0.035; 95%CI = -0.071, 0.003, *P* = 0.04) showed a significant causal relationship. After Benjamini-Hochberg correction, the results indicated a possible causal relationship between ovarian cancer and PA (IVW-FE, *P’* > 0.05) (Fig. 3, Supplementary Figs. 5–6).

### Causal effects of PA on digestive cancers

**Fig. 4:**
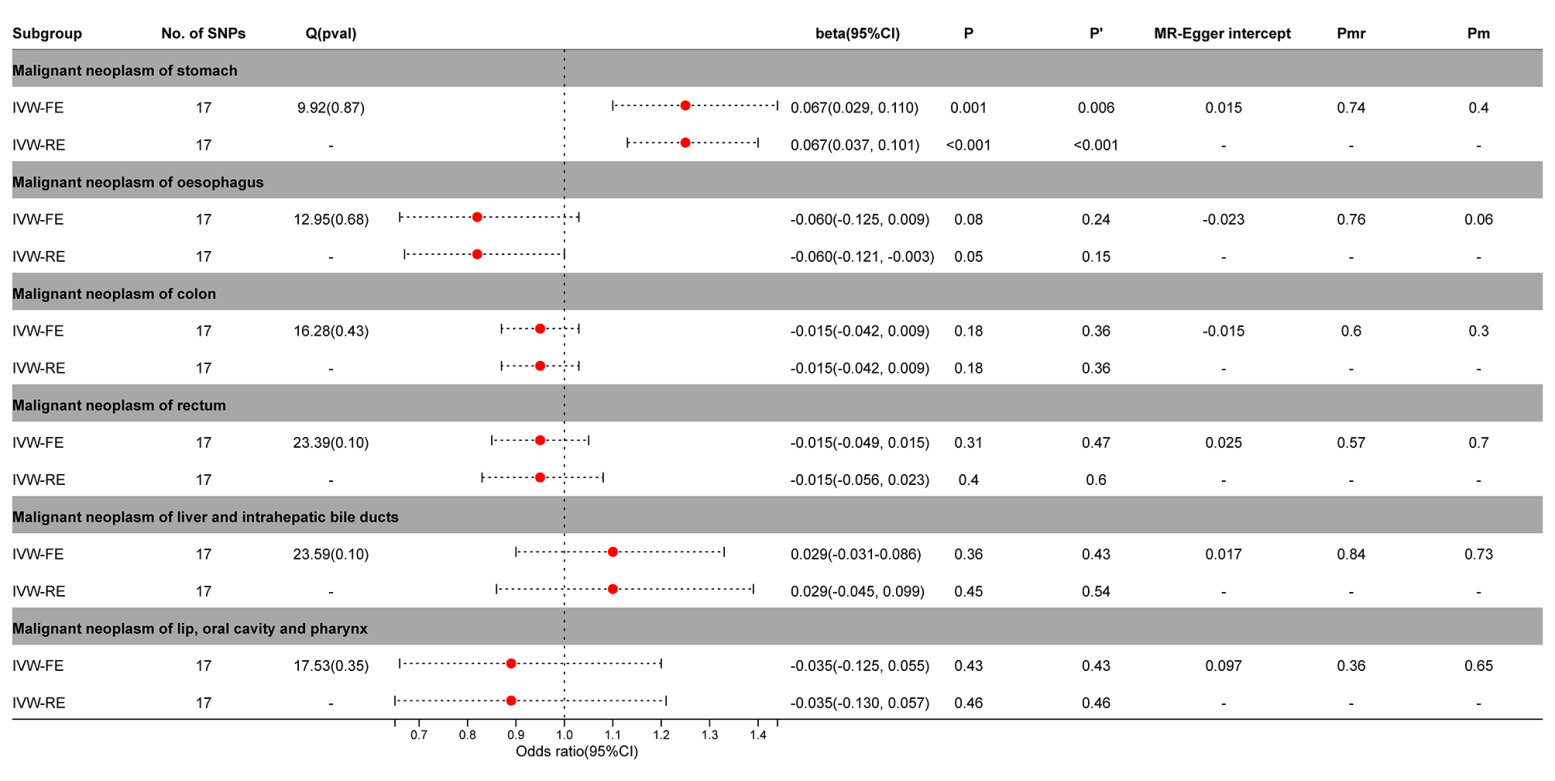
Association between genetic susceptibility to pernicious anemia and digestive cancers. IVW-FE; Inverse Variance Weighted Fixed Effects estimation method; IVW-RE; Inverse Variance Weighted Random Effects estimation method; beta, the average change (when exposed to the variable) in the outcome per 2-fold increase; SNP, single nucleotide polymorphism; 95%CI, 95% Confidence Interval; P, The IVW estimated P-value; P’, The IVW estimated P-value adjusted by the Benjamin-Hochberg method; Q(pval), Cochran Heterogeneity Test P-value; Pmr, MR–Egger P-value; Pm, MRlap P-value; -, Not applicable

The IVW-FE / IVW-RE model before and after correction by Benjamini-Hochberg showed a significant causal relationship between PA and gastric cancer (IVW-FE, beta = 0.067; 95%CI = 0.029, 0.110, *P* = 0.001, *P’* <0.05; IVW-FE, beta = 0.067; 95%CI = 0.037, 0.101, *P* < 0.001, *P’* <0.05) in the MR analysis of digestive tumors and PA (Fig. 4, Supplementary Figs. 8–9).

### Causal effects of PA on other cancers

**Fig. 5:**
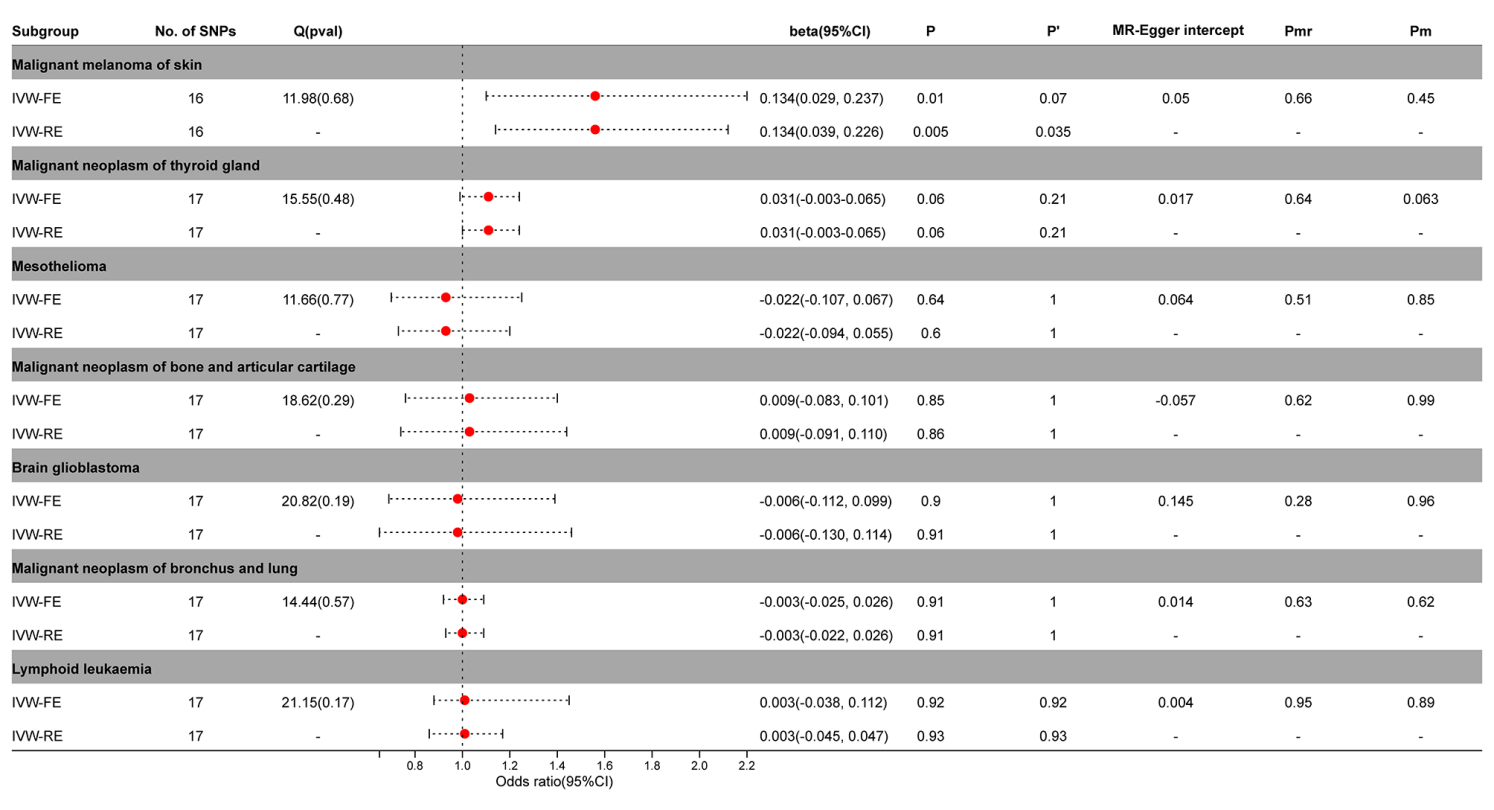
Association between genetic susceptibility to pernicious anemia and other cancers. IVW-FE; Inverse Variance Weighted Fixed Effects estimation method; IVW-RE; Inverse Variance Weighted Random Effects estimation method; beta, the average change (when exposed to the variable) in the outcome per 2-fold increase; SNP, single nucleotide polymorphism; 95%CI, 95% Confidence Interval; P, The IVW estimated P-value; P’, The IVW estimated P-value adjusted by the Benjamin-Hochberg method; Q(pval), Cochran Heterogeneity Test P-value; Pmr, MR–Egger P-value; Pm, MRlap P-value; -, Not applicable

The IVW-FE / IVW-RE model demonstrated a significant causal relationship between PA and malignant melanoma of skin (IVW-FE, beta = 0.134; 95%CI = 0.029, 0.237; *P* = 0.01; IVW-RE, beta = 0.134; 95%CI = 0.039, 0.226; *P* = 0.005)in the MR analysis of PA and other tumors. After correction by Benjamini-Hochberg, the random effects model still supported a significant causal relationship between PA and malignant melanoma of skin (*P* < 0.05), while the fixed effects model only suggested an implied causal relationship (*P* > 0.05) (Fig. 5, Supplementary Figs. 11–12).

### Causal effects of cancers on PA

**Fig. 6:**
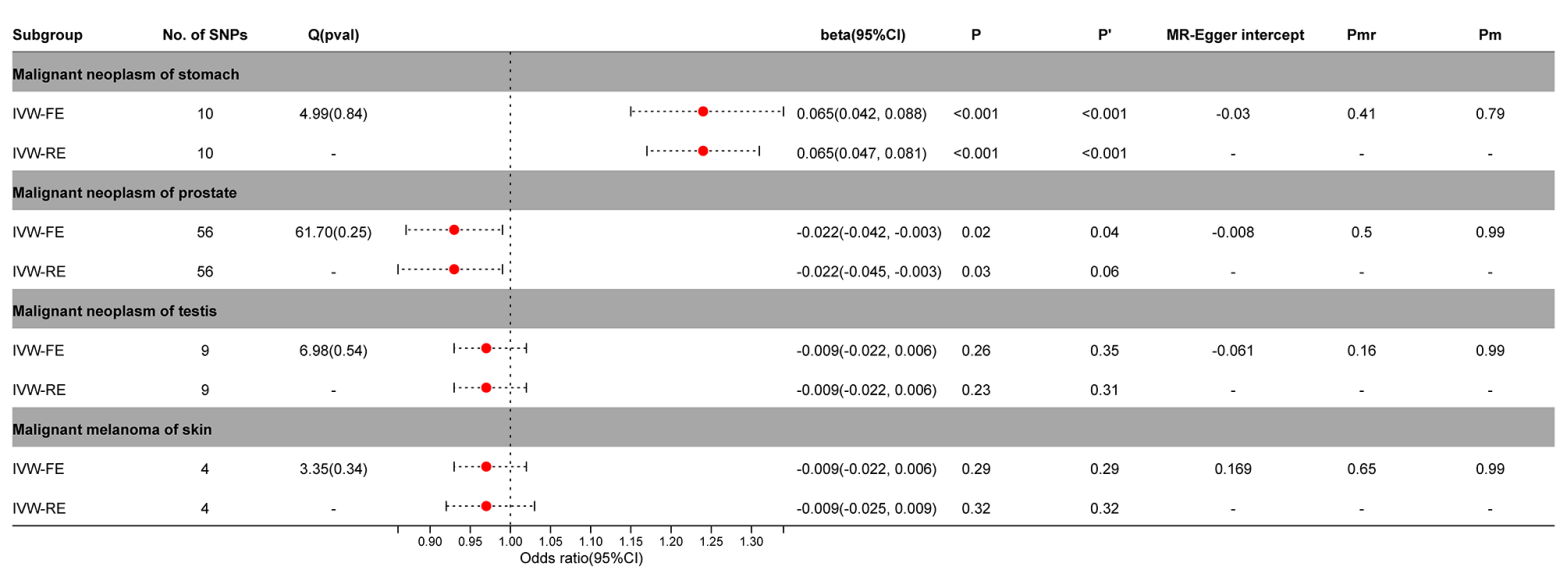
Association between genetic susceptibility to cancers and pernicious anemia. IVW-FE; Inverse Variance Weighted Fixed Effects estimation method; IVW-RE; Inverse Variance Weighted Random Effects estimation method; beta, the average change (when exposed to the variable) in the outcome per 2-fold increase; SNP, single nucleotide polymorphism; 95%CI, 95% Confidence Interval; P, The IVW estimated P-value; P’, The IVW estimated P-value adjusted by the Benjamin-Hochberg method; Q(pval), Cochran Heterogeneity Test P-value; Pmr, MR–Egger P-value; Pm, MRlap P-value; -, Not applicable

We also created a reverse MR to help further elucidate the causal link between PA and malignant melanoma of skin, prostate cancer, gastric cancer, and testicular cancer. The findings demonstrated a significant reverse causal relationship between PA and prostate cancer (IVW-FE, beta =-0.022; 95%CI = -0.042, -0.003; *P* = 0.02; IVW-RE, beta =-0.022; 95%CI = -0.045, -0.003; *P* = 0.03) or gastric cancer (IVW-FE, beta = 0.065; 95%CI = 0.042, 0.088; *P* < 0.001; IVW-RE, beta = 0.065; 95%CI = 0.047, 0.081; *P* < 0.001). After correction by Benjamini-Hochberg, there was still a significant causal relationship between PA and gastric cancer (*P* < 0.05). For prostate cancer, fixed effects model supported a causal relationship with PA (*P* < 0.05), while random effects model had only suggestive effects (*P* > 0.05). Testicular cancer and malignant melanoma of the skin did not exhibit a reverse causal connection with PA (*P* > 0.05) (Fig. 6, Supplementary Figs. 13–15).

## Discussion

PA and subsequent cancer risk have been a focus of attention [6, 10, 11, 20]. However, so far, the association between PA and cancer has been explored only in observational studies for a variety of reasons. Due to the complexity of PA diagnosis, the prevalence of PA is often underestimated in observational studies, so reliable data are not available to assess the subsequent risk of cancer in these patients [12]. It may even get the opposite result. While MR uses the random distribution of genetic variation, its advantage is that it is not disturbed by confounding factors and is not easily affected by reverse causality, which improves the validity of causal inference [14].

This was the first study to investigate the association between PA and 20 site-specific malignancies in the European population using a bidirectional two-sample MR investigation. The findings demonstrated a strong causative link between PA and testicular cancer, prostate cancer, gastric cancer, and malignant melanoma of skin. There was, however, no proof of a connection to the remaining sixteen site-specific malignancies. Reverse MR results showed that prostate cancer and gastric cancer also had causal effects on PA.

PA remains a neglected disease in many healthcare settings and is related to but distinct from autoimmune gastritis. PA occurs in the later stages of autoimmune atrophic gastritis, when gastric factor deficiency and consequent vitamin B12 deficiency may occur [2]. In addition to vitamin B12 deficiency, chronic autoimmune atrophic gastritis is often accompanied by other trace element deficiencies, including vitamin D, etc [21, 22]. Observational studies have confirmed that autoimmune atrophic gastritis is associated with gastric cancer (including gastric neuroendocrine tumors), but the specific pathophysiological mechanism is still unclear. The available evidence supports the relationship between the prevalence of gastric cancer and vitamin D deficiency [23, 24]. The biological function of vitamin D is usually activated by genomic regulation related to VDR in the nucleus or non-genomic action on the cell membrane [25]. After VDR is activated by its antagonist, in most cases, it translocates in the nucleus, regulates the expression of p21, p27 and other target genes, and plays the anticancer effect of vitamin D, including inhibiting cell proliferation and inducing cell apoptosis. In addition to disorders of nutrition and metabolism, it itself acts as an immune system disease [26, 27]。This immune system abnormality may lead to a loss of immune tolerance [28], which may make the body more vulnerable to cancer. In addition, immune system abnormalities may also affect tumor immune surveillance and clearance functions, thereby increasing the risk of cancer. Therefore, the subsequent clinical manifestations of autoimmune atrophic gastritis may be the main reason for increasing the risk of gastric cancer or other cancer.

There is still much controversy about the association between vitamin B12 deficiency and gastric cancer [29]. Vitamin B12 deficiency is characterized by effects on the blood and nervous system. Therefore, the instability of observational studies on the relationship between vitamin B12 and gastric cancer may be related to its complex clinical manifestations, so it may need to be explored for a particular clinical manifestation, such as PA. This may be more helpful in helping people with autoimmune atrophic gastritis / vitamin B12 deficiency benefit from tumor prevention. It is worth noting that, despite the lack of research on PA and cancer, some clinical guidelines still regard PA as a precancerous lesion of gastric cancer and recommend the need for endoscopic monitoring [30, 31]. According to a prior meta-analysis, those with PA had a roughly seven-fold increased relative risk of gastric cancer compared to those without PA [32]. Patients with PA had nearly tripled risk of gastric adenocarcinoma and an 11-fold increased risk of stomach carcinoid tumors, according to another large case-control research [6]. The mechanism underlying the link between PA and the risk of gastric cancer, as well as whether it indicates causation, remain poorly understood, despite the observational studies’ accumulated evidence supporting this association. This may be related to the changes in the environment of the stomach [33]. Due to the lack of gastric acidity caused by the destruction of parietal cells, the environment with high PH may be more conducive to the survival of conditional pathogenic bacteria [34, 35]. Although the exact mechanism is unknown, the results of this study suggest that there is a significant two-way causal relationship between PA patients and gastric cancer, and this MR study can provide additional evidence of a genetic association between PA and gastric cancer.

In addition to digestive tract tumors, the findings provide genetic evidence for a link between PA and androgen-related urinary tumors (prostate and testicular cancer). We speculate that it may be related to the level of vitamin B12. Low levels of vitamin B12 in the body reduced the catalytic activity of methionine synthetase from homocysteine to methionine, resulting in the accumulation of homocysteine in plasma [36]. A number of studies have shown that homocysteine may be related to androgen levels [37, 38]. Therefore, although there is not enough genetic evidence to show that vitamin B12 has a causal relationship with androgen-related urinary tumors [39]. However, previous studies have still observed that vitamin B12 is associated with the risk of prostate cancer [40, 41]. Vitamin B12 supplementation may slow the growth of prostate cancer to some extent [42].

In addition, this study also shows that there was a causal relationship between PA and malignant melanoma of skin. And this is consistent with some previous studies. And a recent large meta-analysis also suggests that PA may increase the risk of malignant melanoma of skin [20]. Therefore, the role of PA in the development of cutaneous melanoma needs more research.

The main advantage of this study is the MR study, which reduces the potential impact of confounding factors compared to observational studies. Secondly, the European sample FinnGen project involved in this MR design has a large sample size to ensure sufficient statistical effectiveness. Finally, we evaluated the association between PA and a variety of cancers for the first time. Of course, there are some limitations. On the one hand, our analysis only covers people of European origin, which, although reducing the impact of race, also limits the universality of our results to other ethnic groups. On the other hand, this study may also be affected by inherent defects in MR analysis, such as the inability to obtain data at the individual level, which may affect the selection bias of IVs. Finally, it is important to emphasize that a binary exposure can lead to violation of the exclusion restriction assumption and limit the inferences drawn from an MR study [43]. Nevertheless, MR remains a robust method to test the causal hypothesis. Therefore, the main purpose of this study was to determine whether a causal relationship exists rather than to estimate the magnitude of the effect.

## Conclusion

In conclusion, this study provides genetic evidence that PA may play a role in the development of several site-specific cancers. Future research is necessary to help us complement cancer prevention strategies.

### Electronic supplementary material

Below is the link to the electronic supplementary material.

Supplementary Material 1

Supplementary Material 2

Supplementary Material 3

Supplementary Material 4

## Data Availability

The datasets analyzed during the current study are available in the FinnGen repository, https://r5.finngen.fi/.
