# Supplementary material for: Causal inference between pernicious anemia and cancers: a bidirectional two-sample mendelian randomization analysis
Source: BMC Cancer. 2024 May 13;24:586. doi: 10.1186/s12885-024-12354-y (PMC11092143; doi:10.1186/s12885-024-12354-y)
Supplement: Supplementary file 1 — Supplementary Material 1 [file 12885_2024_12354_MOESM1_ESM.docx]

Supplementary Table 2. Instrument variables associated with pernicious anemia.

| Chr | SNP | A1 | A2 | Beta | EAF | SE | p | R2 | F |
| --- | --- | --- | --- | --- | --- | --- | --- | --- | --- |
| 1 | rs6679677 | A | C | 0.5190 | 0.1472 | 0.0522 | 2.54E-23 | 4.64E-04 | 98.8539 |
| 2 | rs1990760 | T | C | 0.2093 | 0.5850 | 0.0358 | 5.09E-09 | 1.61E-04 | 34.1800 |
| 2 | rs17292123 | G | A | 0.3605 | 0.0611 | 0.0749 | 1.50E-06 | 1.09E-04 | 23.1658 |
| 3 | rs17656368 | T | C | -0.1633 | 0.5620 | 0.0356 | 4.56E-06 | 9.89E-05 | 21.0413 |
| 3 | rs7634746 | G | C | 0.1801 | 0.3270 | 0.0378 | 1.90E-06 | 1.07E-04 | 22.7009 |
| 3 | rs35896106 | T | C | 0.3026 | 0.0800 | 0.0661 | 4.69E-06 | 9.85E-05 | 20.9573 |
| 6 | rs9321367 | T | G | -0.2548 | 0.1276 | 0.0536 | 2.02E-06 | 1.06E-04 | 22.5980 |
| 6 | rs28407950 | T | C | -0.2863 | 0.3439 | 0.0422 | 1.16E-11 | 2.16E-04 | 46.0275 |
| 6 | rs9270535 | A | G | -0.2202 | 0.4247 | 0.0395 | 2.42E-08 | 1.46E-04 | 31.0771 |
| 6 | rs140650994 | C | G | 2.5099 | 0.0014 | 0.5472 | 4.50E-06 | 9.88E-05 | 21.0388 |
| 8 | rs79132259 | T | G | 0.4866 | 0.0333 | 0.1024 | 1.99E-06 | 1.06E-04 | 22.5811 |
| 10 | rs75973258 | G | A | 0.7672 | 0.0127 | 0.1677 | 4.75E-06 | 9.83E-05 | 20.9291 |
| 12 | rs7310615 | G | C | -0.1710 | 0.5862 | 0.0357 | 1.71E-06 | 1.08E-04 | 22.9433 |
| 16 | rs151234 | C | G | 0.3049 | 0.1242 | 0.0544 | 2.05E-08 | 1.48E-04 | 31.4136 |
| 19 | rs35056955 | G | A | 1.0305 | 0.0079 | 0.2215 | 3.28E-06 | 1.02E-04 | 21.6446 |
| 19 | rs73597298 | G | T | -0.3088 | 0.1099 | 0.0577 | 8.58E-08 | 1.35E-04 | 28.6420 |
| 21 | rs2826921 | A | C | 0.2174 | 0.2214 | 0.0426 | 3.32E-07 | 1.22E-04 | 26.0435 |
