# Supplementary material for: Causal inference between pernicious anemia and cancers: a bidirectional two-sample mendelian randomization analysis
Source: BMC Cancer. 2024 May 13;24:586. doi: 10.1186/s12885-024-12354-y (PMC11092143; doi:10.1186/s12885-024-12354-y)
Supplement: Supplementary file 2 — Supplementary Material 2 [file 12885_2024_12354_MOESM2_ESM.docx]

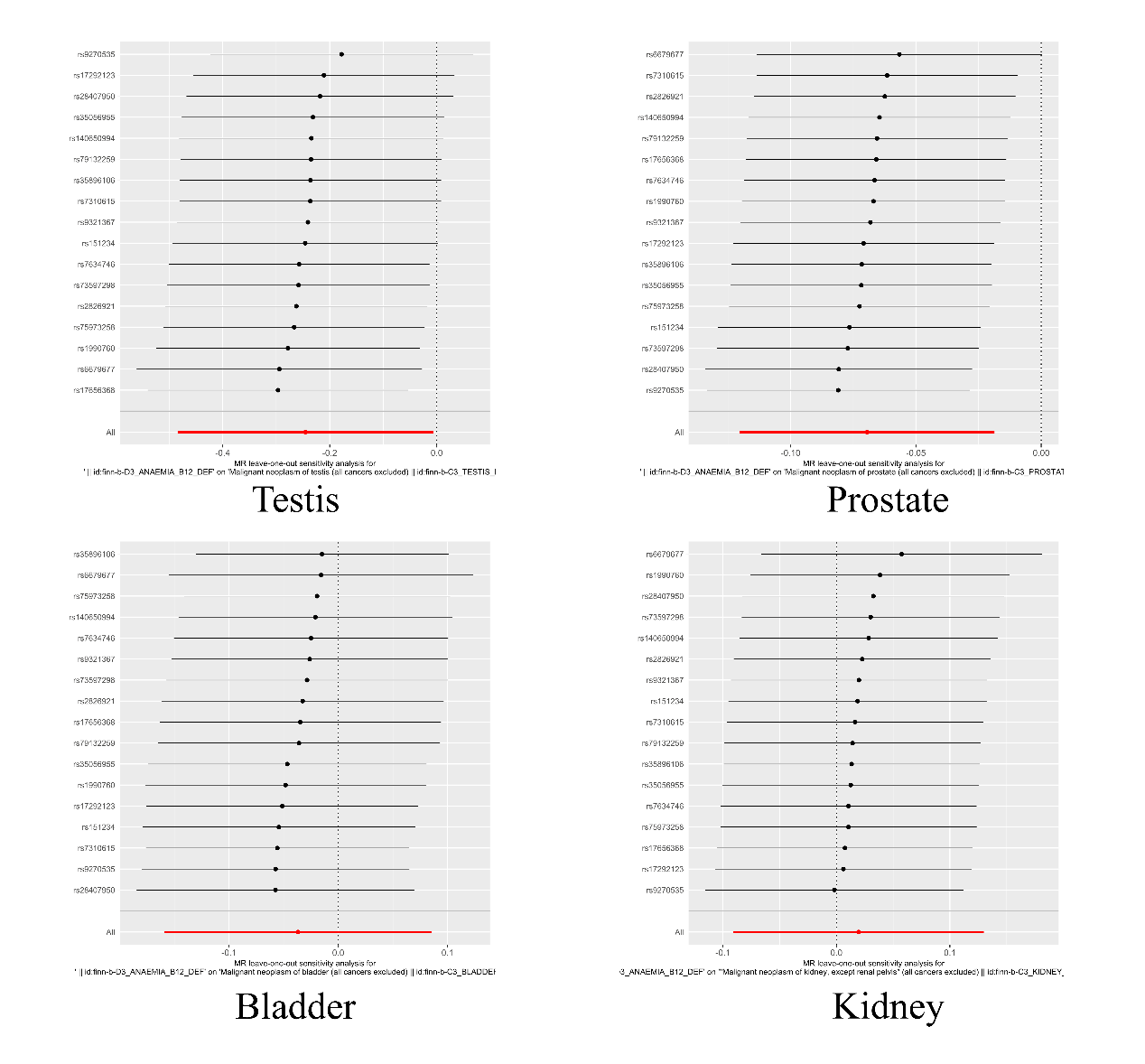


Supplementary figure 1. The leave-one-out plot of SNPs associated with pernicious anemia and their risk on urinary cancers.


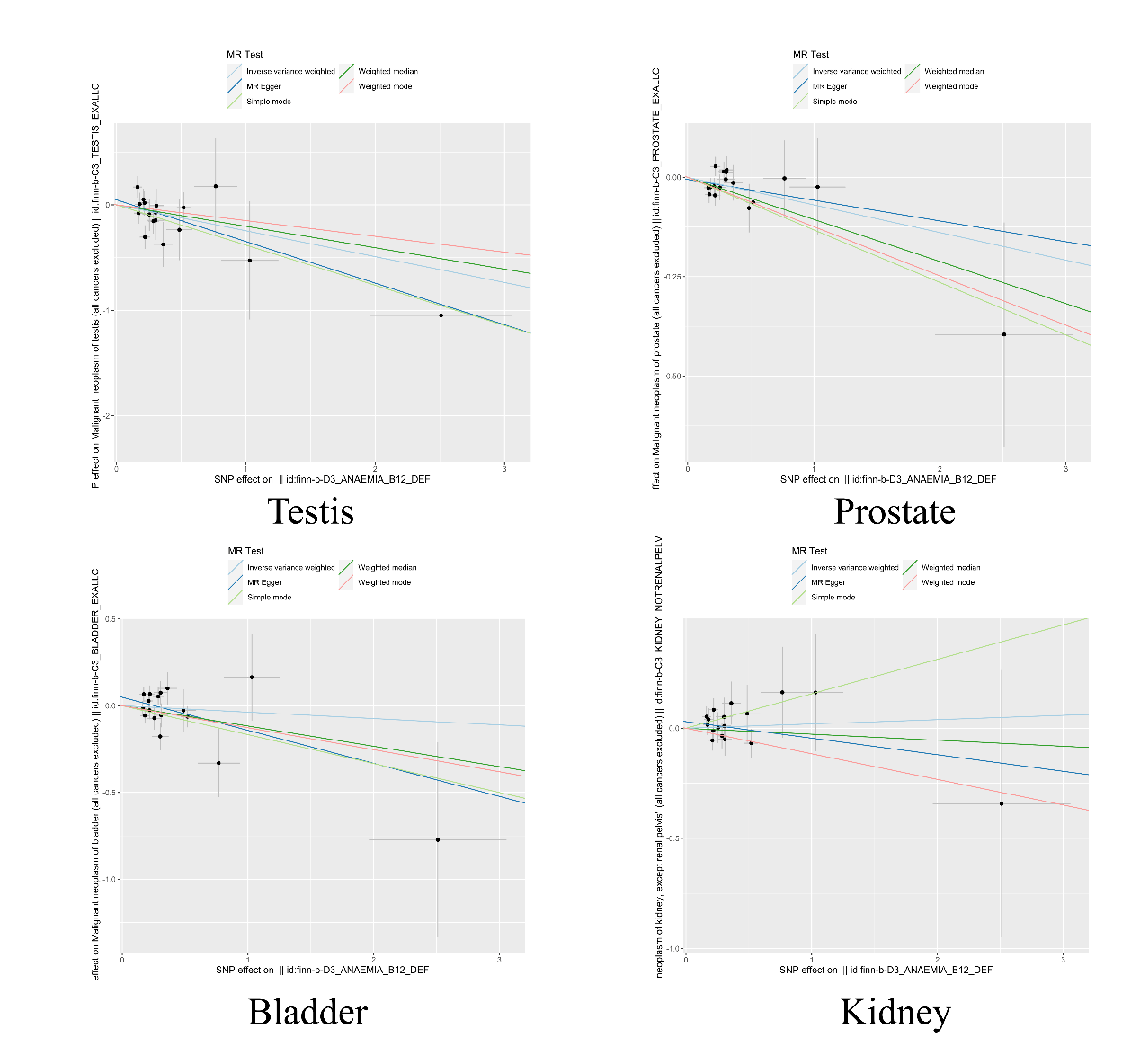


Supplementary figure 2. Scatter plots of SNPs associated with pernicious anemia and their risk on urinary cancers.


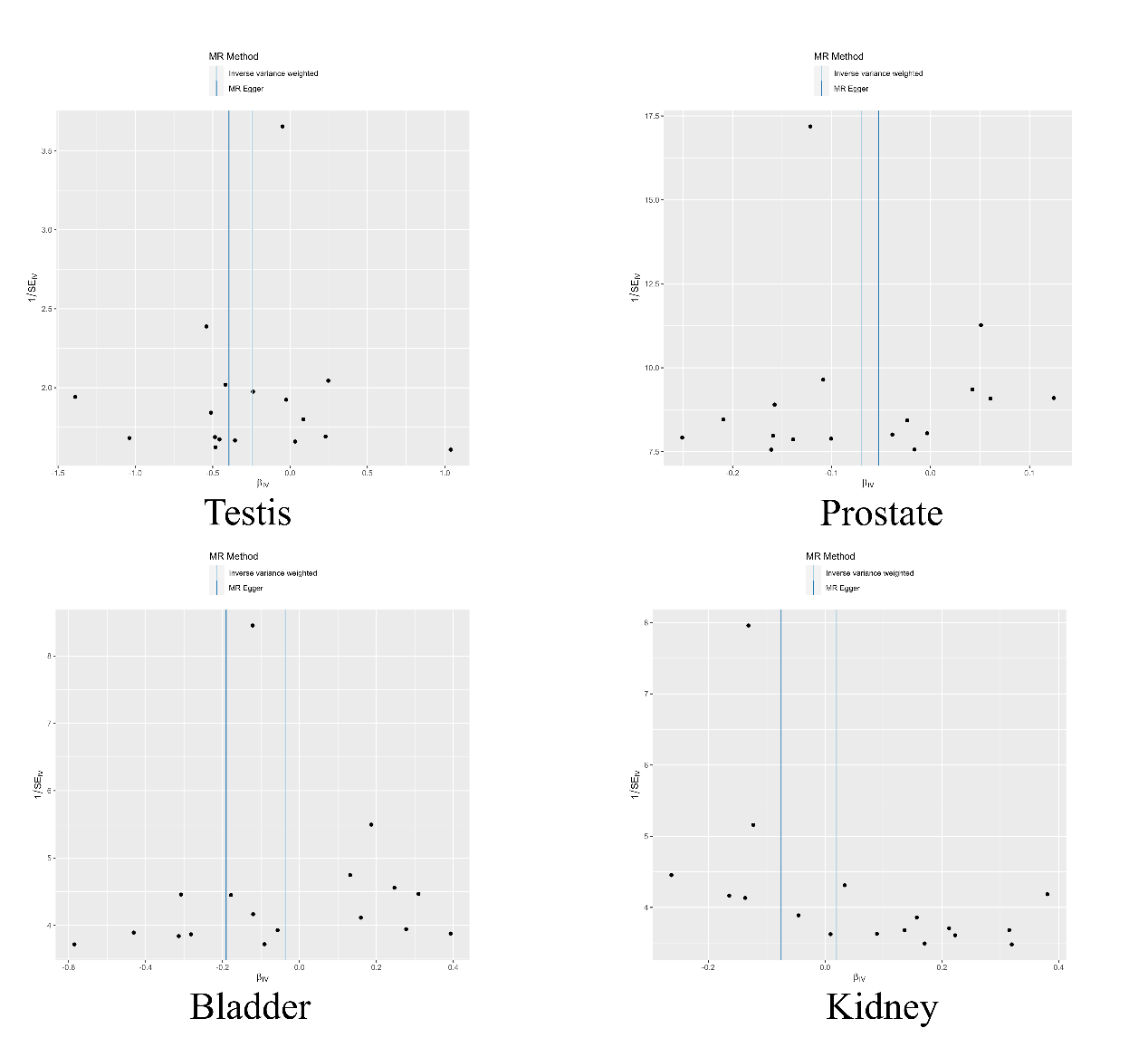


Supplementary figure 3. Funnel plots of SNPs associated with pernicious anemia and their risk on urinary cancers.


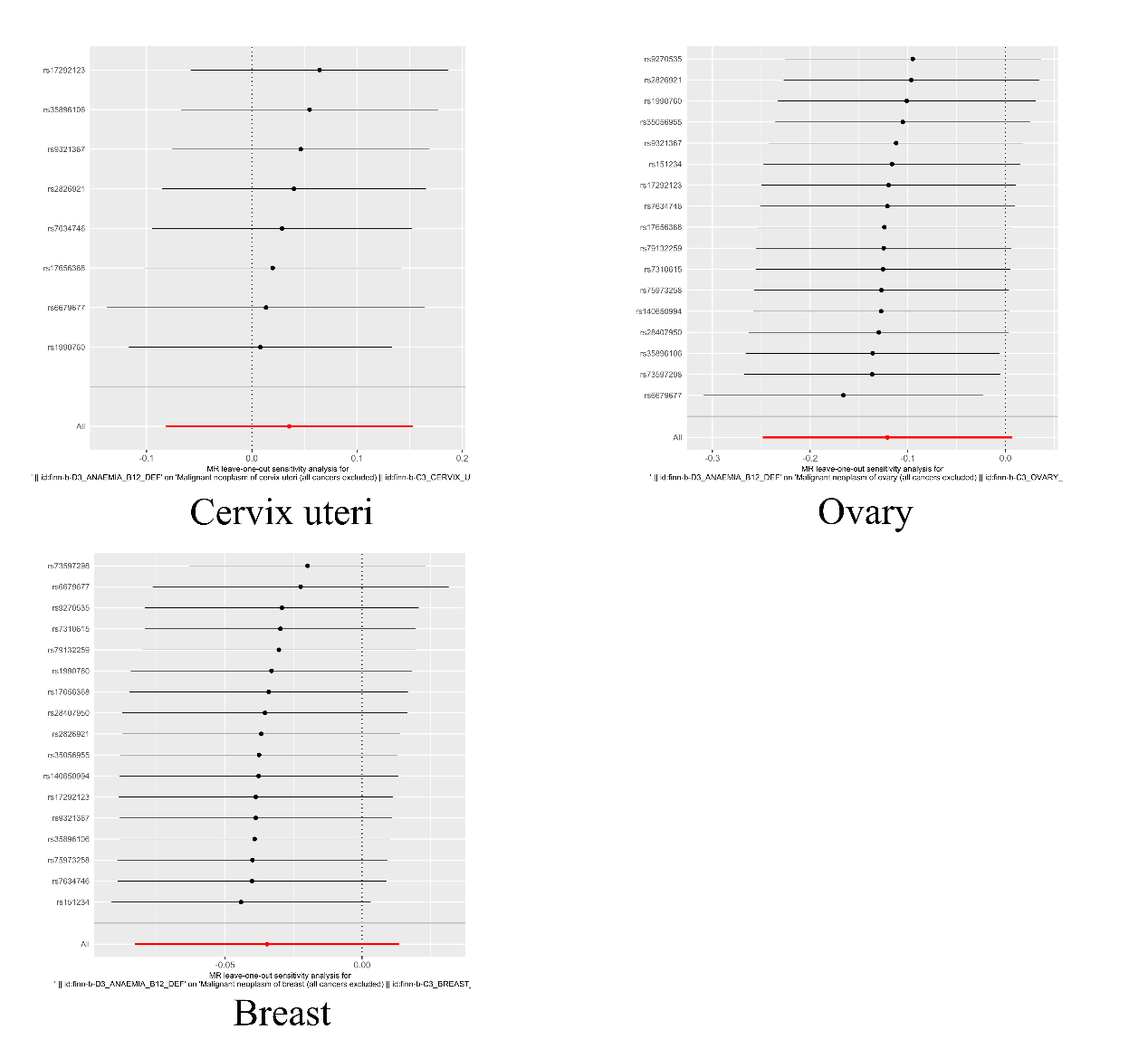


Supplementary figure 4. The leave-one-out plot of SNPs associated with pernicious anemia and their risk on female cancers.


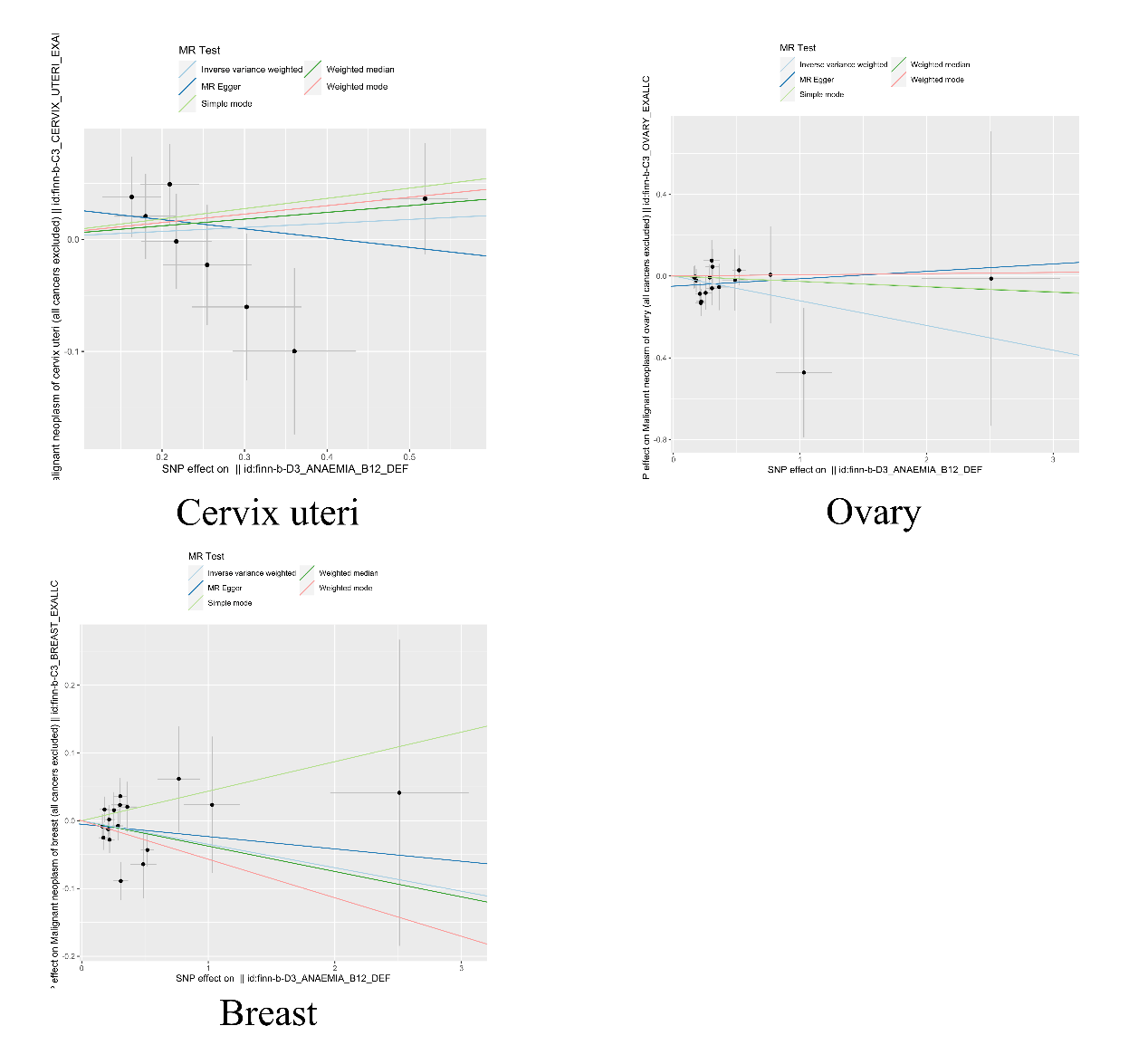


Supplementary figure 5. Scatter plots of SNPs associated with pernicious anemia and their risk on female cancers.


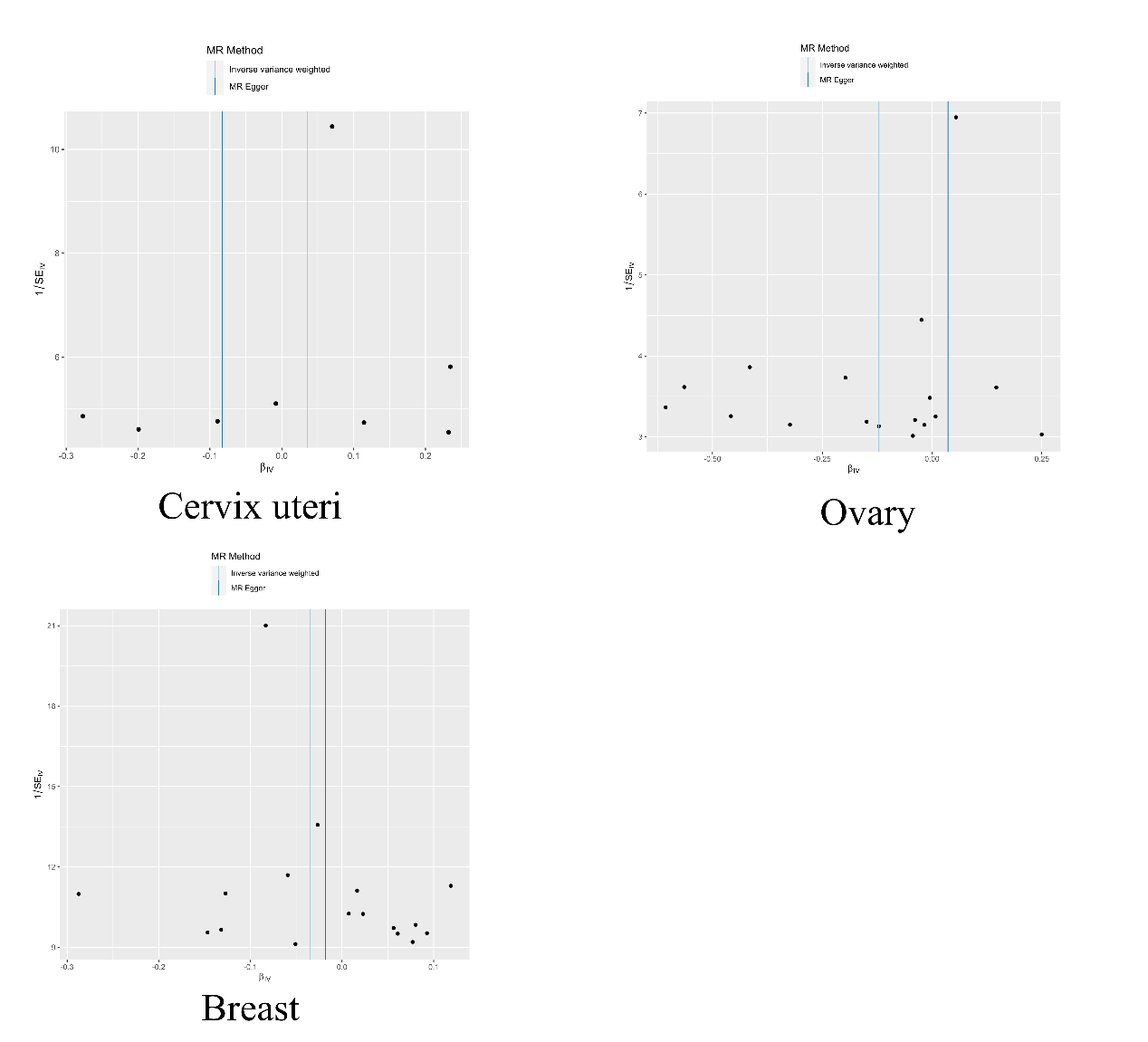


Supplementary figure 6. Funnel plots of SNPs associated with pernicious anemia and their risk on female cancers.


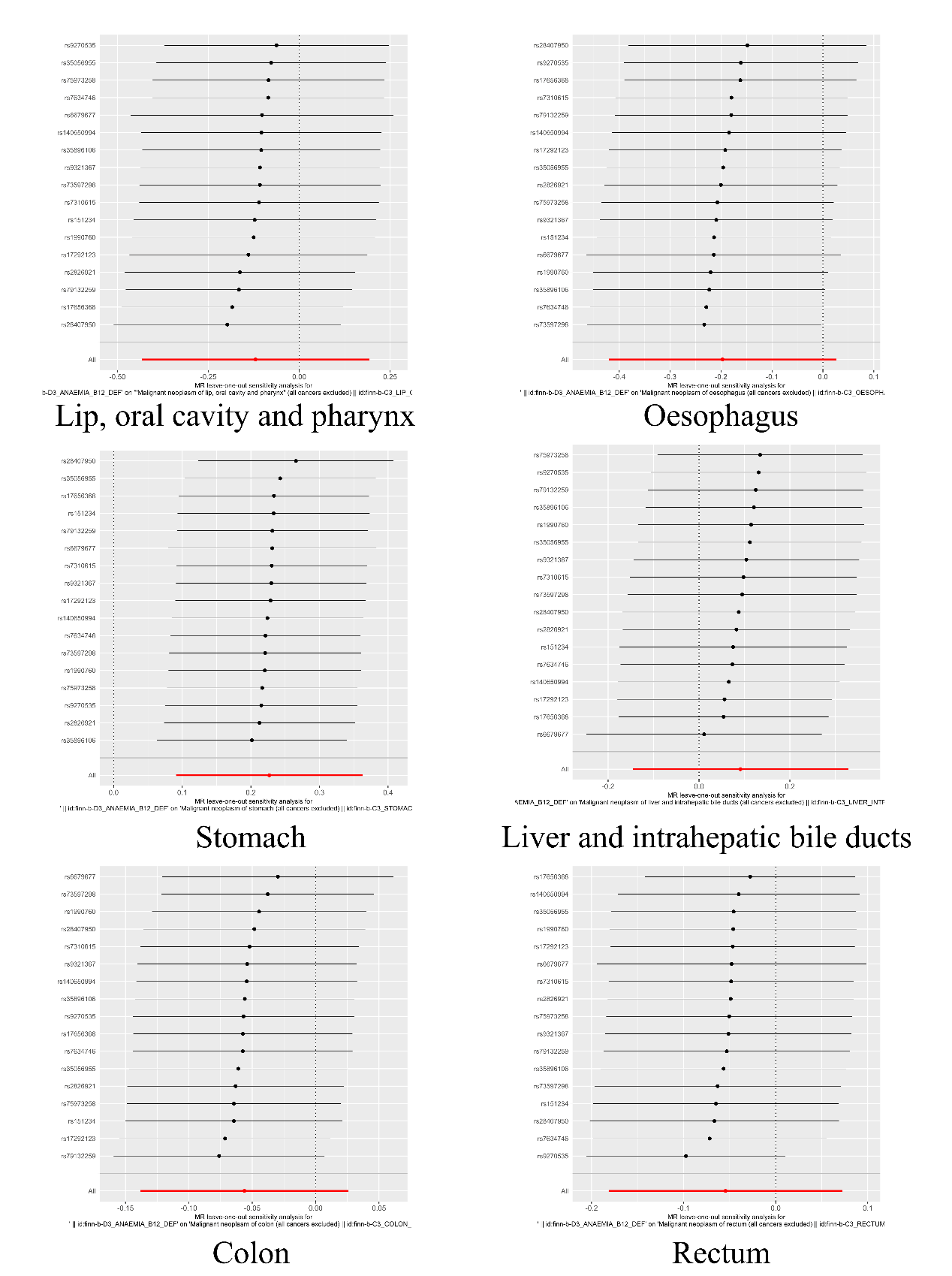


Supplementary figure 7. The leave-one-out plot of SNPs associated with pernicious anemia and their risk on digestive cancers.


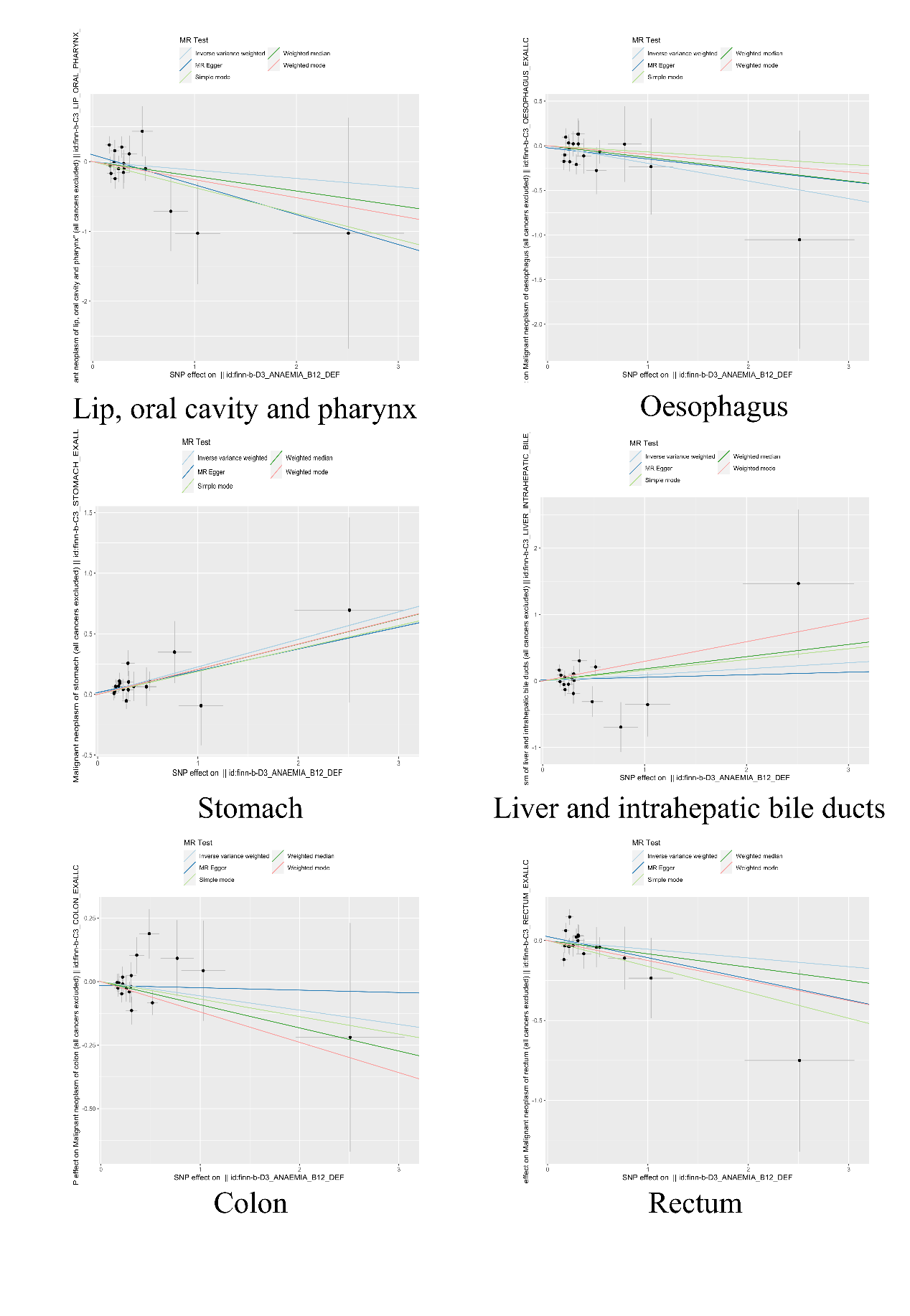


Supplementary figure 8. Scatter plots of SNPs associated with pernicious anemia and their risk on digestive cancers.


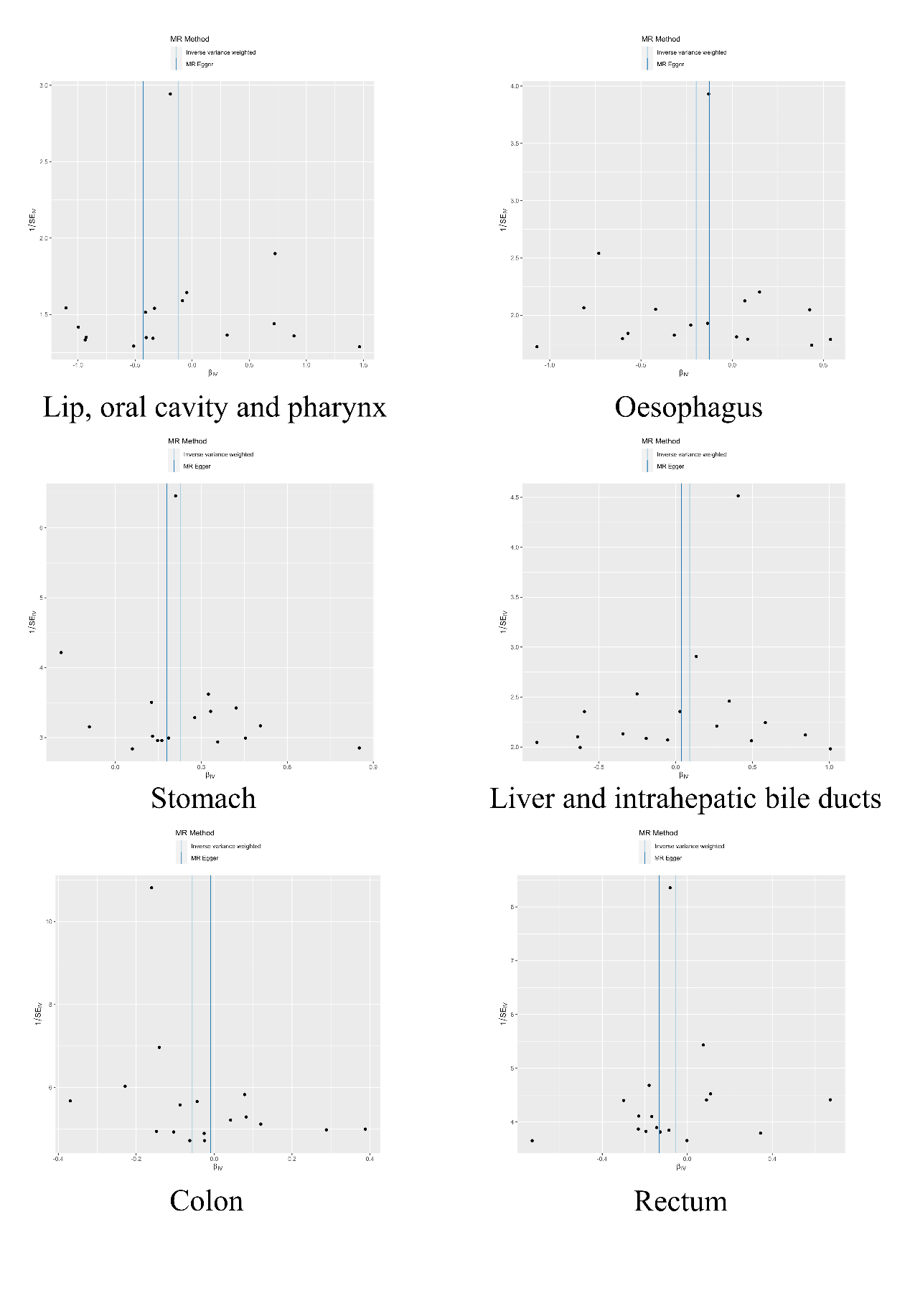


Supplementary figure 9. Funnel plots of SNPs associated with pernicious anemia and their risk on digestive cancers.


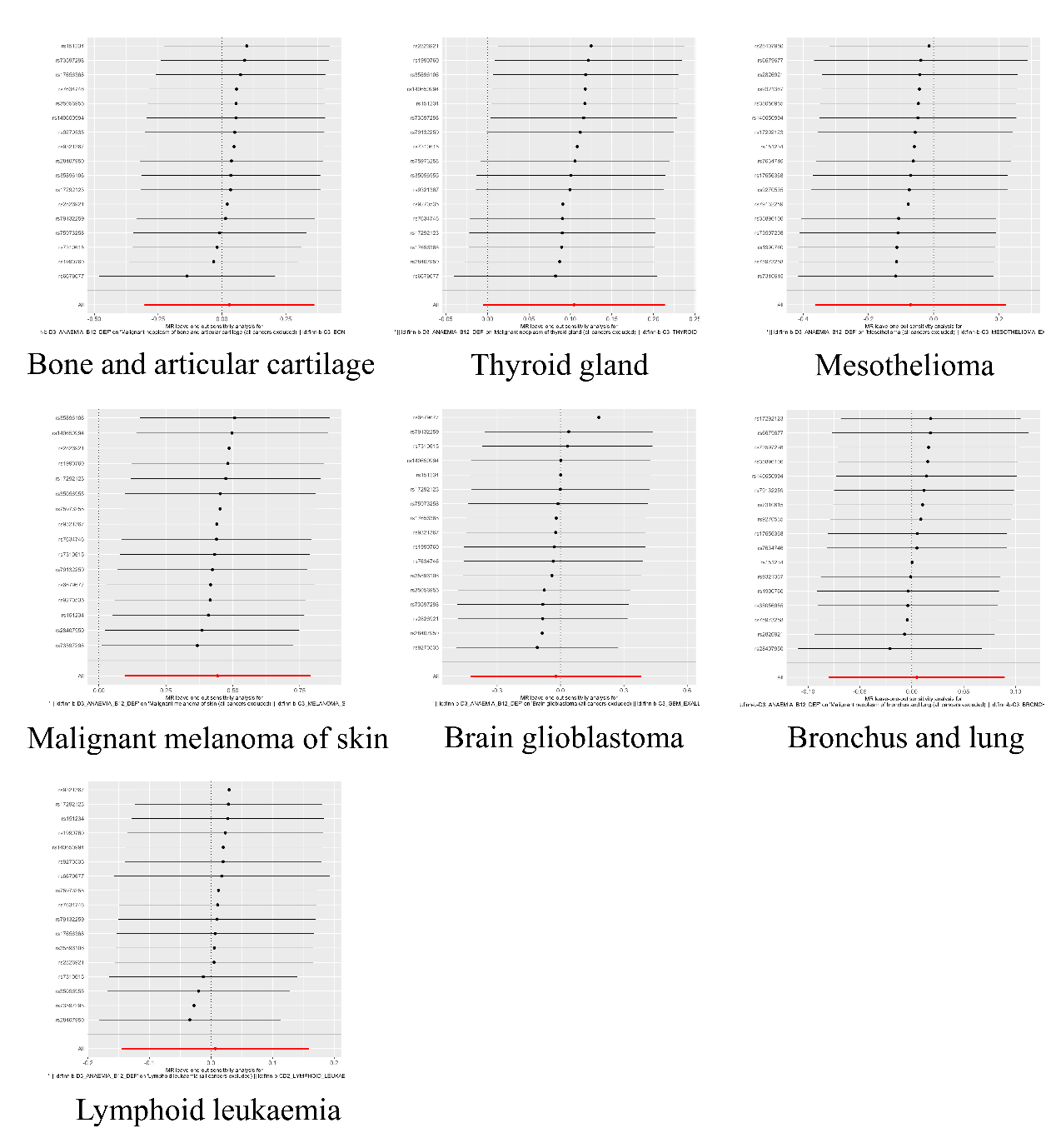


Supplementary figure 10. The leave-one-out plot of SNPs associated with pernicious anemia and their risk on other cancers.


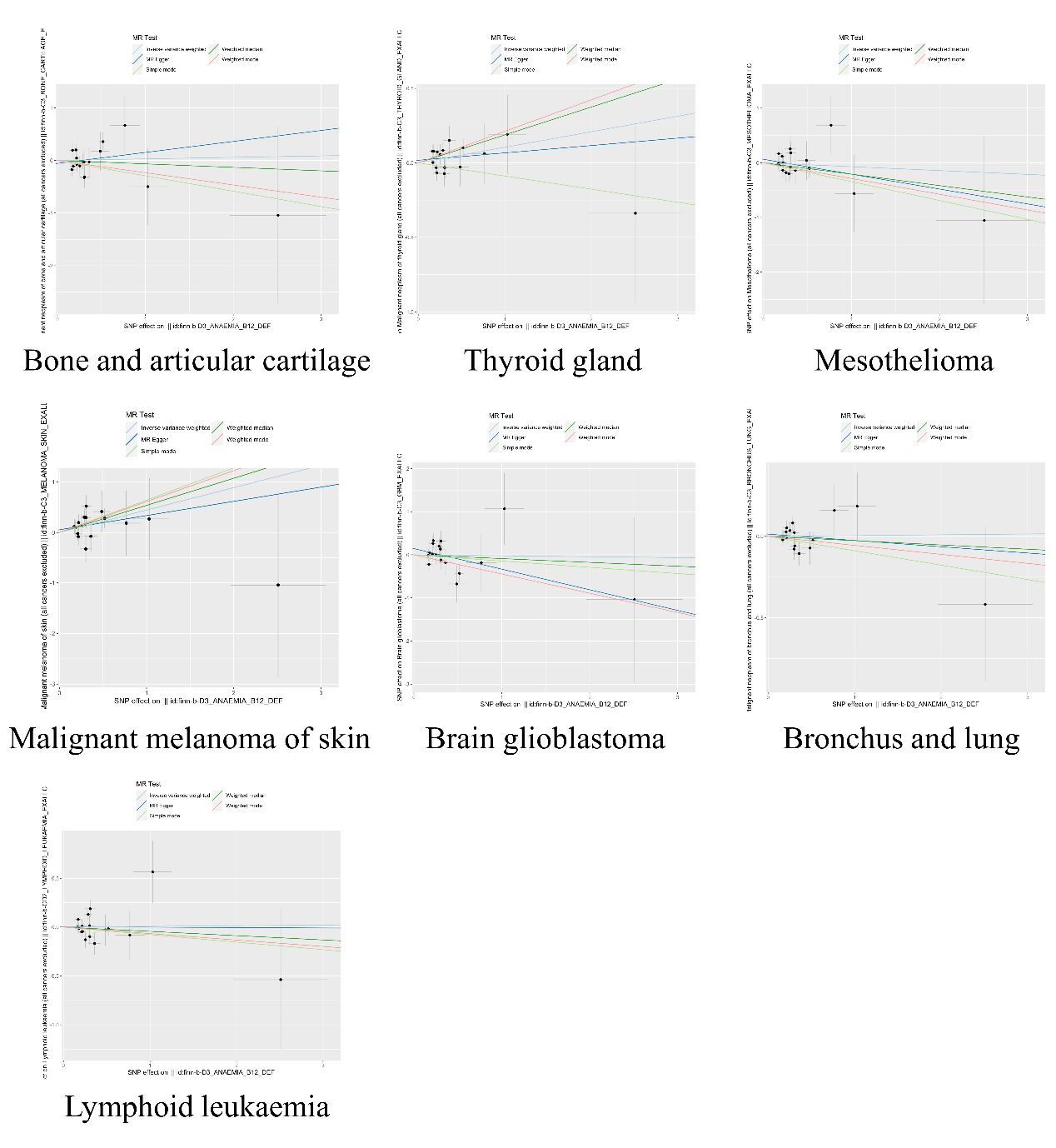


Supplementary figure 11. Scatter plots of SNPs associated with pernicious anemia and their risk on other cancers.


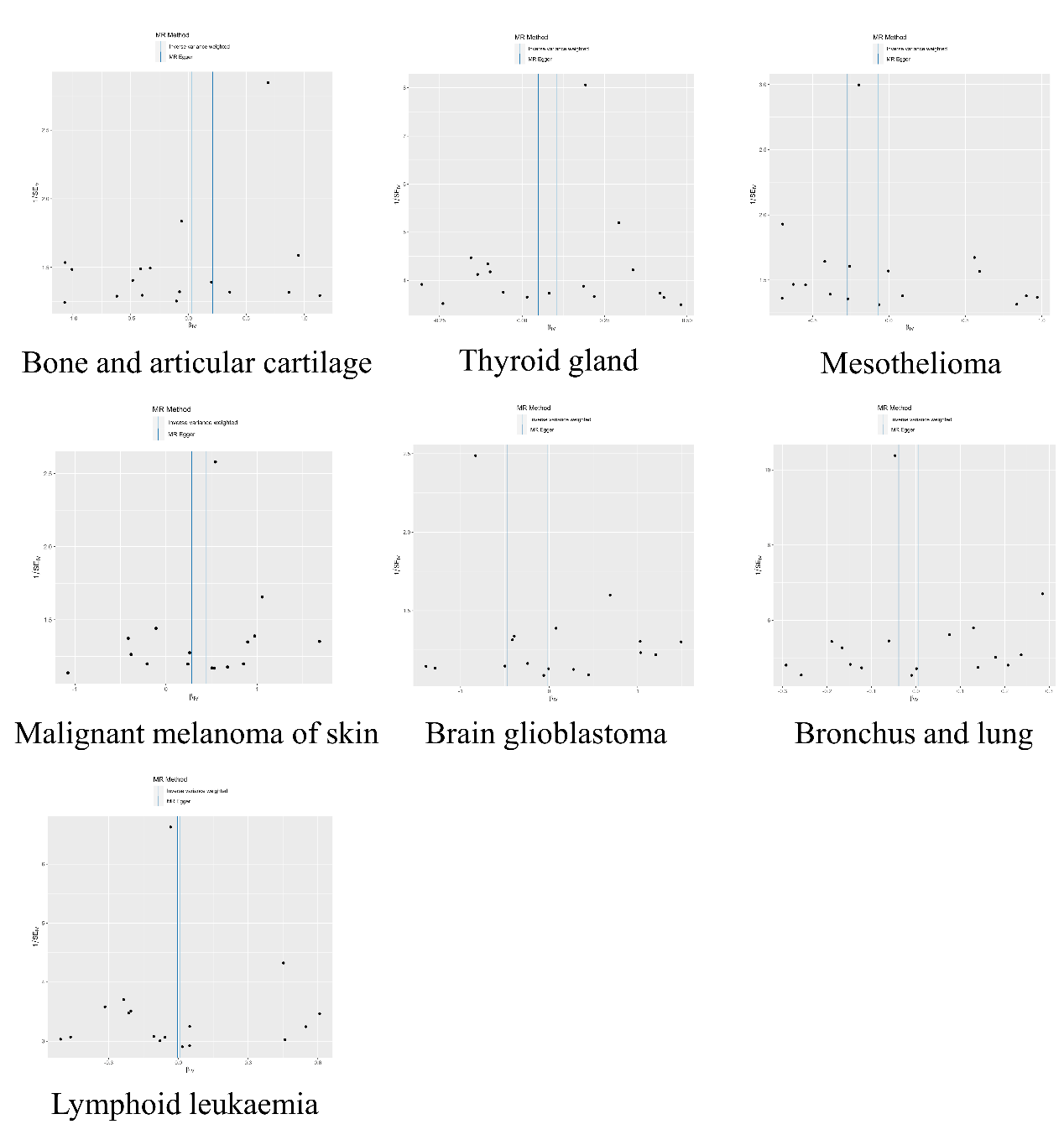


Supplementary figure 12. Funnel plots of SNPs associated with pernicious anemia and their risk on other cancers.


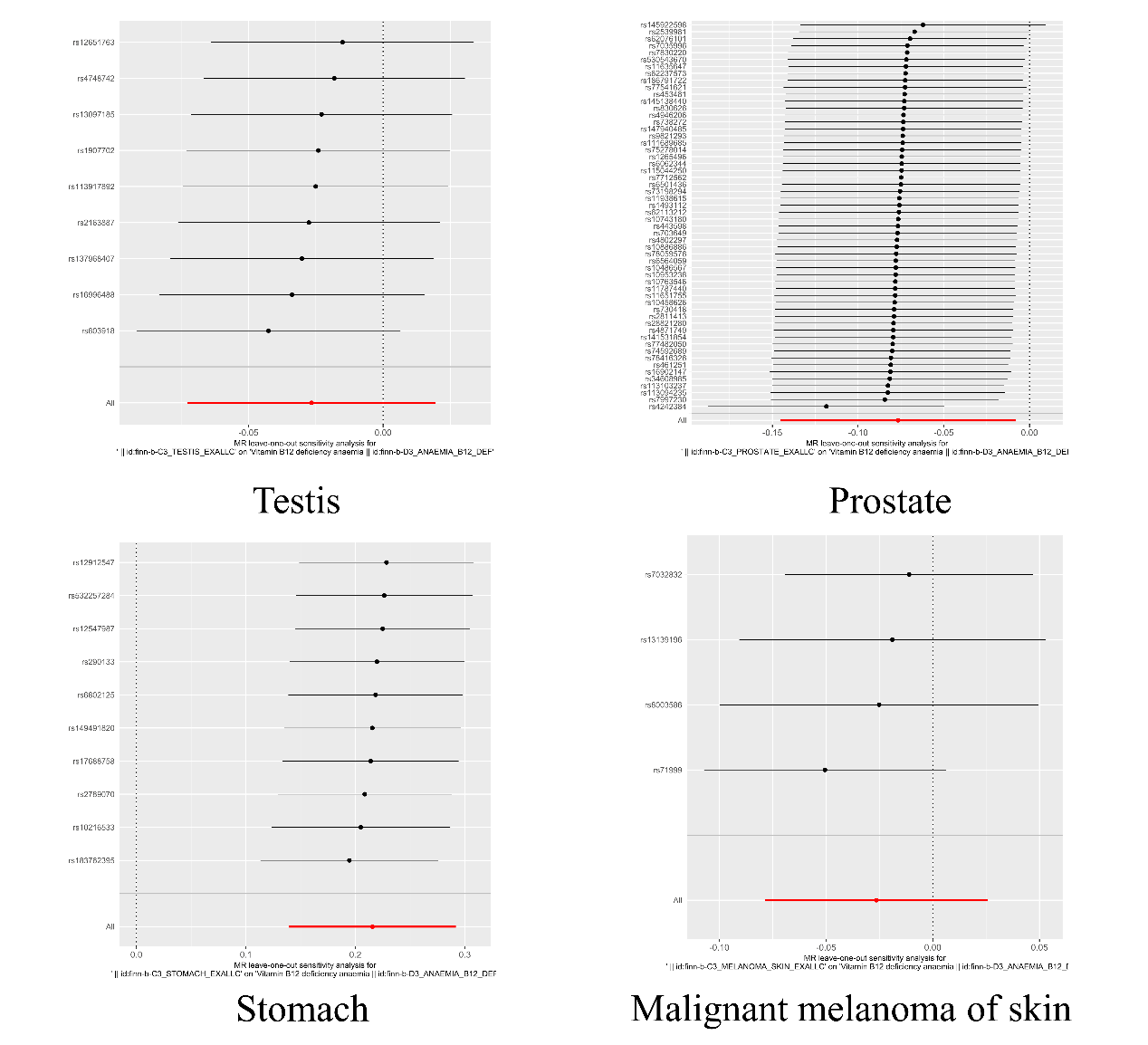


Supplementary figure 13. The leave-one-out plot of SNPs associated with cancers and their risk on pernicious anemia.


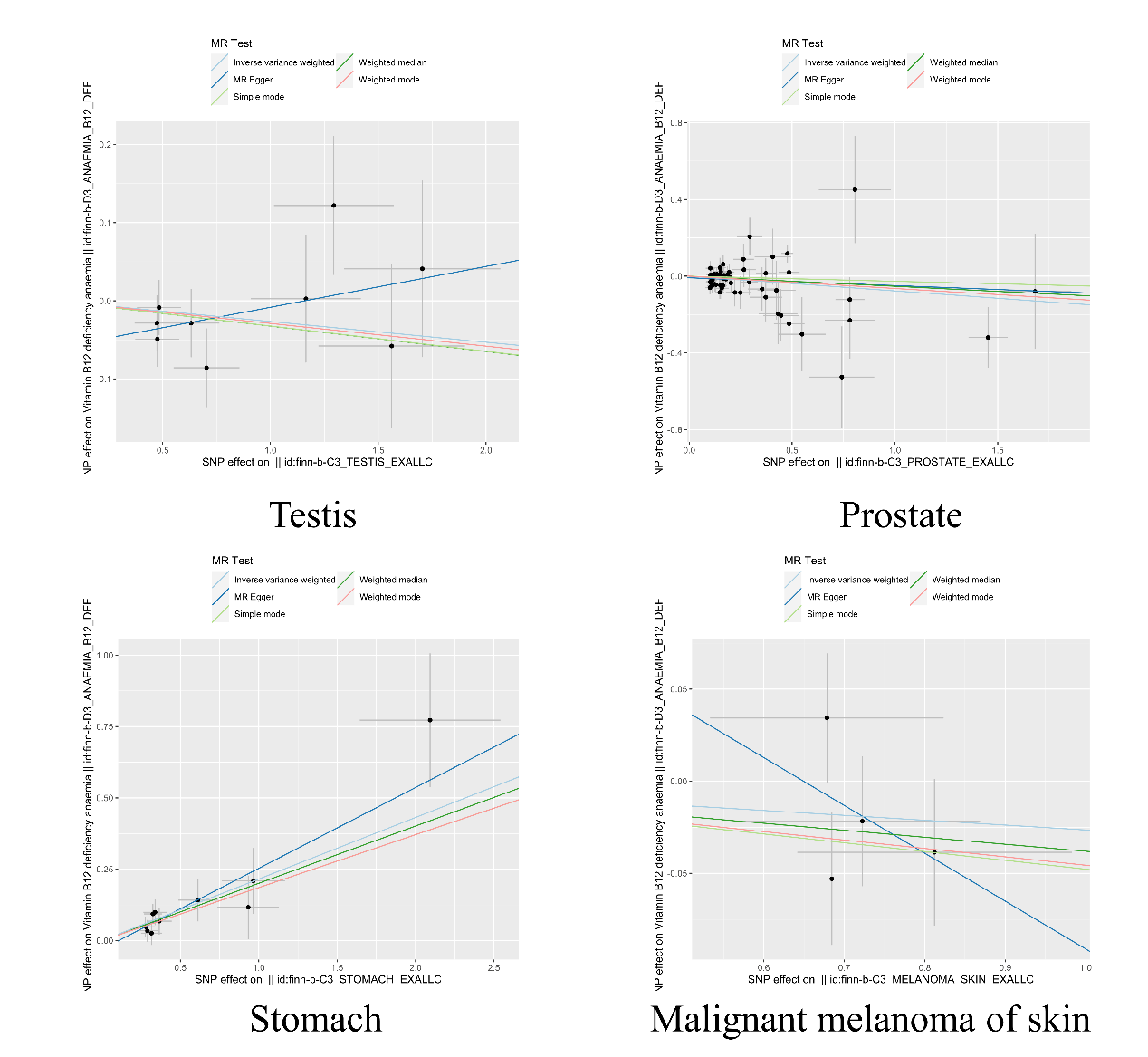


Supplementary figure 14. Scatter plots of SNPs associated with cancers and their risk on pernicious anemia.


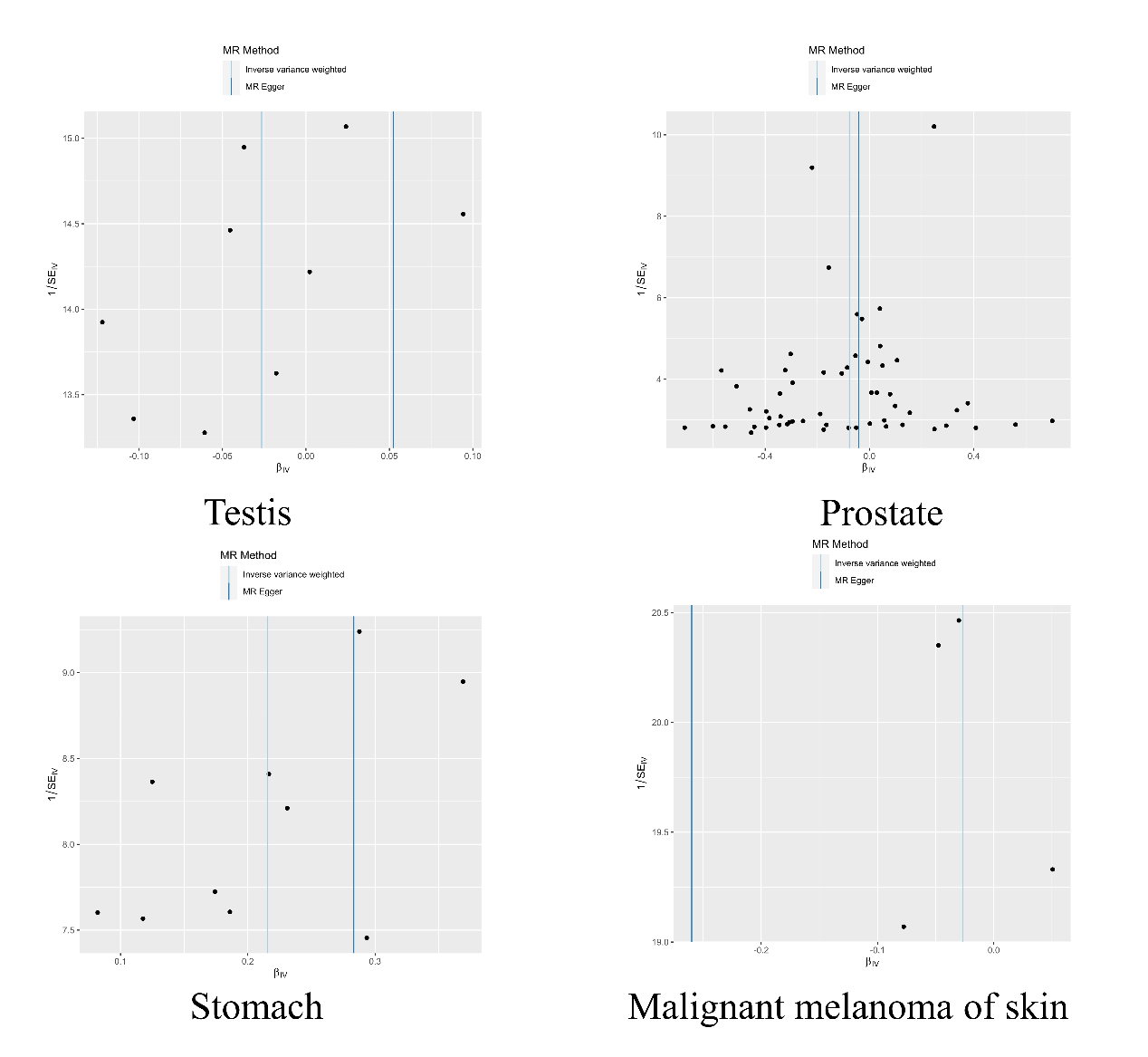


Supplementary figure 15. Funnel plots of SNPs associated with cancers and their risk on pernicious anemia.
