# Supplementary material for: Causal inference between pernicious anemia and cancers: a bidirectional two-sample mendelian randomization analysis
Source: BMC Cancer. 2024 May 13;24:586. doi: 10.1186/s12885-024-12354-y (PMC11092143; doi:10.1186/s12885-024-12354-y)
Supplement: Supplementary file 3 — Supplementary Material 3 [file 12885_2024_12354_MOESM3_ESM.docx]

Supplementary Table 1. Source of of genome-wide association study summary data from

| Exposure/outcomes | Study/Consortium | Cases | Controls | No. of SNPs | Years | Population |
| --- | --- | --- | --- | --- | --- | --- |
| Vitamin B12 deficiency anaemia | FinnGen | 1,707 | 211,115 | 16,380,452 | 2021 | European |
| Malignant neoplasm of prostate | FinnGen | 6,311 | 74,685 | 16,377,987 | 2021 | European |
| Malignant neoplasm of ovary | FinnGen | 719 | 99,321 | 16,378,913 | 2021 | European |
| Malignant neoplasm of bronchus and lung | FinnGen | 1,681 | 173,993 | 16,380,305 | 2021 | European |
| Malignant neoplasm of oesophagus | FinnGen | 232 | 174,006 | 16,380,304 | 2021 | European |
| Malignant neoplasm of liver and intrahepatic bile ducts | FinnGen | 304 | 174,006 | 16,380,303 | 2021 | European |
| Malignant neoplasm of kidney | FinnGen | 971 | 174,006 | 16,380,308 | 2021 | European |
| Malignant neoplasm of stomach | FinnGen | 633 | 174,006 | 16,380,305 | 2021 | European |
| Malignant neoplasm of colon | FinnGen | 1,803 | 174,006 | 16,380,317 | 2021 | European |
| Malignant neoplasm of bladder | FinnGen | 1,115 | 174,006 | 16,380,305 | 2021 | European |
| Malignant neoplasm of breast | FinnGen | 8,401 | 99,321 | 16,379,328 | 2021 | European |
| Malignant neoplasm of cervix uteri | FinnGen | 1,648 | 99,321 | 16,378,927 | 2021 | European |
| Malignant neoplasm of rectum | FinnGen | 1,078 | 174,006 | 16,380,307 | 2021 | European |
| Lymphoid leukaemia | FinnGen | 663 | 180,756 | 16,380,339 | 2021 | European |
| Brain glioblastoma | FinnGen | 91 | 174,006 | 16,380,303 | 2021 | European |
| Mesothelioma | FinnGen | 133 | 174,006 | 16,380,303 | 2021 | European |
| Malignant melanoma of skin | FinnGen | 98 | 174,006 | 16,380,303 | 2021 | European |
| Malignant neoplasm of testis | FinnGen | 199 | 74,685 | 16,377,430 | 2021 | European |
| Malignant neoplasm of thyroid gland | FinnGen | 989 | 174,006 | 16,380,316 | 2021 | European |
| Malignant neoplasm of bone and articular cartilage | FinnGen | 119 | 174,006 | 16,380,303 | 2021 | European |
| Malignant neoplasm of lip, oral cavity and pharynx | FinnGen | 126 | 174,006 | 16,380,304 | 2021 | European |
