# Supplementary material for: Causal inference between pernicious anemia and cancers: a bidirectional two-sample mendelian randomization analysis
Source: BMC Cancer. 2024 May 13;24:586. doi: 10.1186/s12885-024-12354-y (PMC11092143; doi:10.1186/s12885-024-12354-y)
Supplement: Supplementary file 4 — Supplementary Material 4 [file 12885_2024_12354_MOESM4_ESM.docx]

Supplementary Table 3. Results of MR-PRESSO.

| **Exposure / Outcome** | **SNP outliers** | ***P*** |
| --- | --- | --- |
| **Outcome** |  |  |
| Malignant neoplasm of prostate | - | 0.599 |
| Malignant neoplasm of testis | - | 0.517 |
| Malignant neoplasm of bladder | - | 0.128 |
| Malignant neoplasm of kidney | - | 0.801 |
| Malignant neoplasm of ovary | - | 0.681 |
| Malignant neoplasm of breast | - | 0.199 |
| Malignant neoplasm of cervix uteri | rs9270535, rs140650994, rs79132259, rs75973258, rs7310615, rs151234, rs35056955, rs73597298 | 0.571 |
| Malignant neoplasm of stomach | - | 0.874 |
| Malignant neoplasm of oesophagus | - | 0.694 |
| Malignant neoplasm of colon | - | 0.448 |
| Malignant neoplasm of rectum | - | 0.113 |
| Malignant neoplasm of liver and intrahepatic bile ducts | - | 0.103 |
| Malignant neoplasm of lip, oral cavity and pharynx | - | 0.383 |
| Malignant melanoma of skin | - | 0.728 |
| Malignant neoplasm of thyroid gland | - | 0.778 |
| Mesothelioma | - | 0.778 |
| Malignant neoplasm of bone and articular cartilage | - | 0.266 |
| Brain glioblastoma | - | 0.168 |
| Malignant neoplasm of bronchus and lung | - | 0.580 |
| Lymphoid leukaemia | - | 0.188 |
| **Exposure** |  |  |
| Malignant neoplasm of stomach | - | 0.862 |
| Malignant neoplasm of prostate | - | 0.208 |
| Malignant neoplasm of testis | - | 0.533 |
| Malignant melanoma of skin | - | 0.375 |
